# The Relationship Between Physical Activity and Mental Health Among Individuals With Spinal Cord Injury: Protocol for a Scoping Review

**DOI:** 10.2196/56081

**Published:** 2024-06-12

**Authors:** Winslet Ong, Noor Arfa Omar, Asfarina Zanudin, Muhamad Faiz Alias, Lim Hui Wen, Angel Thang Xue Ee, Nor Azlin Mohd Nordin, Haidzir Manaf, Basri Husin, Mahadir Ahmad, Hafifi Hisham

**Affiliations:** 1 Pusat Rehabilitasi PERKESO Tun Abdul Razak Melaka Malaysia; 2 Center for Rehabilitation and Special Needs Studies Faculty of Health Sciences Universiti Kebangsaan Malaysia Kuala Lumpur Malaysia; 3 Institut Latihan Kementerian Kesihatan Malaysia Sungai Buloh Malaysia; 4 Center of Physiotherapy Faculty of Health Sciences Universiti Teknologi MARA Puncak Alam Malaysia; 5 Malaysian Spinal Cord Injury Advocacy Association Putrajaya Malaysia; 6 Faculty of Health Sciences Universiti Kebangsaan Malaysia Kuala Lumpur Malaysia

**Keywords:** access barriers, depression, disability, exercise interventions, mental health, mobility limitations, physical activity, psychological outcomes, quality of life, spinal cord injury, SCI, mobility, scoping review, exercise, effectiveness, mental health, well-being, impairments, physical function, disability, prevalence

## Abstract

**Background:**

Spinal cord injury (SCI) is a devastating condition that often leads to significant impairments in physical function, leading to disability and mental health disorders. Hence, understanding the prevalence of SCI and the relationship between physical activity and mental health in individuals with SCI is crucial for informing rehabilitation strategies and optimizing outcomes.

**Objective:**

This study aims to comprehensively analyze existing research on the link between physical activity and mental health and identify the level of physical activity and mental health status, the barriers to physical activity, and SCI’s impacts on psychological well-being in individuals with SCI.

**Methods:**

An electronic search strategy will be used to identify prevalence studies published since 1993 in health-related databases such as PubMed, MEDLINE, COCHRANE Library, and Wiley Library using the following query: “Spinal Cord Injury” OR “Paraplegia” OR “Tetraplegia” AND “Physical Activity” OR “Exercise” AND “Mental Health” OR “Mental Illness” OR “Mental Disorder.” Bibliographies of primary studies and review articles meeting the inclusion criteria will be searched manually to identify further eligible studies. The risk of bias in the included studies will be appraised using the Joanna Briggs Institute checklist for prevalence studies by 2 review authors. Any disagreement will be resolved by reaching a consensus.

**Results:**

Funding was received in October 2023, data collection will commence in July 2024, and the results are expected by 2025. We will summarize the selection of the eligible studies using a flowchart. The data from the studies will be extracted and tabulated. This scoping review will be published in a peer-reviewed journal in accordance with PRISMA-ScR (Preferred Reporting Items for Systematic Reviews and Meta-Analyses extension for Scoping Reviews) guidelines.

**Conclusions:**

This scoping review underscores the complex relationship between physical activity and mental health among individuals with SCI, highlighting the level of physical activity and mental health status, barriers to physical activity engagement, and psychological implications. Understanding these dynamics is crucial in devising tailored interventions aimed at enhancing mental well-being. This synthesis of evidence emphasizes the need for personalized strategies to promote physical activity, addressing unique challenges faced by this population to foster improved mental health outcomes and overall quality of life.

**Trial Registration:**

Open Science Framework osf.io/ugx7d; https://osf.io/ugx7d/

**International Registered Report Identifier (IRRID):**

PRR1-10.2196/56081

## Introduction

Spinal cord injury (SCI) is a devastating event that often leads to significant issues with physical function and mobility and is defined by varying degrees of sensory and motor dysfunction [1,2]. SCI can result from traumatic events such as motor vehicle accidents [3,4], falls [4,5], sports and recreation [6], or nontraumatic events such as degenerative disc diseases [7,8], cancer, myelopathy [9], and other diseases [10]. Individuals with SCI face numerous daily obstacles, including pain [11], neurogenic bowel and bladder [12], restrictions in strength, endurance, cardiovascular fitness, flexibility, and functional mobility, which affect physiological function [13,14], their return to work [15], and quality of life (QOL) [16].

Physical activity is any movement that requires energy expenditure and engages our musculature. Regular physical activity provides numerous advantages, including improved cardiovascular health, increased strength, and flexibility [17], improved mental well-being, weight management, and a decreased risk of chronic diseases such as diabetes and hypertension [18,19]. Depression is an example of a mental health disorder characterized by persistent sadness, loss of interest or delight in activities, and a spectrum of emotional and physical symptoms [20,21]. It can affect daily functioning, employment [22], and QOL [23].

A review has been conducted to determine the effects of physical activity on mental health after SCI [24]. However, this review’s findings have limited generalizability to broader populations or settings [25,26] and difficulty in establishing causality [27] compared to a review of prevalence studies. Therefore, a review of prevalence studies to determine the level of physical activity and mental health as well as their association, including the barrier and psychological impacts, is essential and useful particularly for conducting future intervention studies or trials and informing health care professionals, researchers, policy makers, and rehabilitation specialists to improve the QOL and care for individuals with SCI. By providing a holistic overview, this study seeks to bridge gaps in understanding and informing more personalized rehabilitation strategies, thereby enhancing the overall QOL for individuals with SCI. The rationale for this study lies in the necessity to comprehensively understand the relationship between physical activity and mental health among individuals with SCI. The objectives of this study are to (1) determine the association between physical activity and mental health level, (2) address the level of physical activity and mental health status, and (3) identify barriers to physical activity and their impact on psychological well-being as well as QOL after SCI.

## Methods

### Search Strategy

The first author WO will conduct a scoping review in accordance with PRISMA-ScR (Preferred Reporting Items for Systematic Reviews and Meta-Analysis extension for Scoping Reviews) guidelines with a literature search using MeSH (Medical Subject Headings) terms in various health-related databases such as PubMed, COCHRANE Library, MEDLINE, Web of Science, and the Wiley Online Library (January 1993 to December 2023). A starting point of 1993 will be selected for exploring a wide array of research findings and trends, providing valuable insights into the evolution of knowledge and practices in the field over the past 3 decades. Databases such as the aforementioned ones were selected for their comprehensive coverage of biomedical and health-related research. PubMed and MEDLINE offer extensive coverage of peer-reviewed biomedical literature, while COCHRANE Library specializes in clinical trials. In addition, a bibliography and gray literature search will be performed to locate additional potential studies that are already relevant. Bibliography searching, also known as citation searching or reference chaining, involves examining the reference lists of relevant articles that may not have been captured during the initial database search. The gray literature search will be conducted in the ProQuest Dissertations & Theses Global database.

The search terms or keywords used were based on the population, such as “Spinal Cord Injury” OR “Paraplegia” OR “Tetraplegia” and outcomes such as “Physical Activity” OR “Exercise” AND “Mental Health” OR “Mental Illness” OR “Mental Disorder.” The elements from the databases were then combined using the Boolean operator (Table 1).

**Figure 1:**
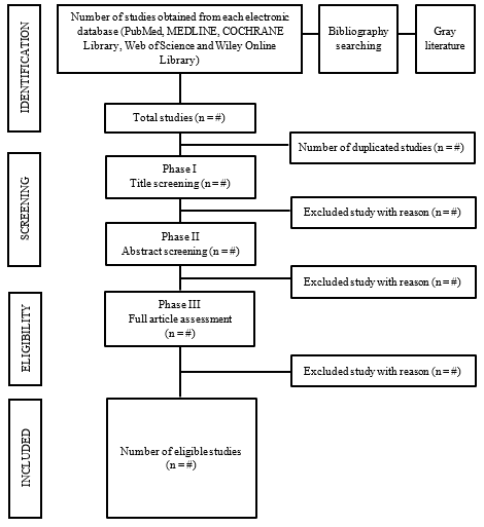
PRISMA-ScR (Preferred Reporting Items for Systematic Reviews and Meta-Analyses extension for Scoping Reviews) flowchart of study searching and selection to obtain eligible studies to be included in the review of mental health outcomes among individuals with spinal cord injury.

The inquiry was restricted to studies published in the English language from 1993 to 2023 and involving only human participants. The flowchart of the study is shown in Figure 1. The search will be modified for each database, and the obtained references will be managed using the EndNote reference management software (Clarivate).

**Table 1:** Search strategies to obtain eligible studies on mental health among individuals with spinal cord injury.

Criteria	Databases
Databases	PubMed, MEDLINE, COCHRANE Library, Web of Science, and Wiley Online Library
Bibliography searching	Reference lists of relevant articles
Gray literature	ProQuest Dissertations & Theses Global
Keywords	(Spinal Cord Injury OR Paraplegia OR Tetraplegia) AND (Physical Activity OR Exercise) AND (Mental Health OR Mental Illneses OR Mental Disorder)
Limiters	Humans, English language, adults (19-65 years old), 1993, 2003, and academic journals
Search modes	Boolean/Phrase

### Selection Criteria

#### Inclusion Criteria

Studies will be included if they were published between January 1993 and December 2023 and if they are cross-sectional, prospective, or retrospective cohort studies, and any other study designs except for randomized or nonrandomized controlled trials, systematic or scoping reviews, and meta-analyses, qualitative studies, regardless of age, gender, ethnicity, severity of injury, level of injury, socioeconomic status, level of education, socioeconomic, or study setting.

#### Exclusion Criteria

To maintain the integrity and relevance of the review by ensuring that only studies with well-defined objectives and methodologies are included, studies will be excluded if they do not report the primary outcome and do not provide a clear description of the case definition, full-text articles are unavailable, full-text access is unavailable, and are animal studies. Additionally, systematic reviews and meta-analyses will be excluded because they represent secondary analyses rather than primary research studies. In this scoping review, qualitative data will be excluded from the analysis. The decision to exclude qualitative data is based on the scope and objectives of the review, which will primarily focus on synthesizing quantitative evidence to address the research question effectively. By excluding qualitative data, the review aims to maintain a clear and focused approach, ensuring that the synthesis process remains manageable and aligned with the predetermined research objectives.

### Outcomes

Our outcomes include the participants’ characteristics, studies’ characteristics, and risk-of-bias assessment.

### The Review and Data Extraction Process

Inclusion and exclusion criteria will be used to determine the admissibility of studies. The 3-phase filtration procedure will commence with the title (first phase), followed by the abstract (second phase), and finally the full text (third phase). Duplicate studies will be eliminated. Author WO will screen and select pertinent research from the database and the reference lists of selected articles for inclusion in the review. Authors WO and NAO will extract data on the demographic characteristics of the study populations, including age, gender, years since injury, level and severity of injury, and type of injury, ethnicity, province or district, study setting (community or health facility-based), setting (urban or rural), sample size, response rate, and prevalence of physical capacity. The following data will also be extracted: study title, study design, duration, objective, intervention, and outcomes. After completion, the data will be compared, and any discrepancies will be resolved through a consensus discussion between the 2 examiners or in consultation with a third reviewer.

The data extraction process will involve systematically collecting relevant information from each included study to address the research objectives. Key variables to be extracted include (1) demographic characteristics (age, gender, and severity of injury), (2) study characteristics related to physical activity and mental health (study design, intervention details, and outcome measures), (3) the level of physical activity and mental health status, (4) the level of association between physical activity and mental health, (5) barriers or facilitators to participation in physical activity among individuals with SCI. Additionally, data on the prevalence of physical activity and mental health outcomes will be recorded. To handle missing data, a pragmatic approach will be adopted. Initially, attempts will be made to contact study authors for additional information or clarification regarding missing data. If missing data are to be obtained, author WO will document the missing data and the steps taken to address them in the final review report.

### Assessment of Risk of Bias

A checklist created by the Joanna Briggs Institute (JBI) will be used to evaluate the quality of methodology of each prevalence study to determine the extent to which each study addressed the possibility of bias in its design, conduct, and analysis [28]. The JBI Scientific Committee authorized the JBI checklist after a thorough peer review. The JBI checklist has high face validity and acceptability levels, is easy to administer, and requires less evaluation time. In addition, the JBI checklist is presently being evaluated for its other clinometric properties, including construct validity and interrater reliability, as part of a larger-scale study. The JBI checklist uses the following criteria to evaluate studies: (1) a representative sample; (2) an appropriate apparatus; (3) an adequate sample size; (4) an appropriate description and study report, subjects, and setting; (5) adequate data coverage; (6) measurement of the condition’s validity; (7) measurement of the condition’s dependability; (8) appropriate statistical analysis; and (9) the adequacy of the response rate. To assess studies for low or moderate risk of bias, reviewers examine each criterion based on predefined thresholds or criteria. A study with a representative sample that adequately reflects the target population may be considered as having a low risk of bias, while a study with significant sampling limitations may be classified as having a moderate risk of bias. Two evaluators will evaluate the eligible studies, and any disagreements will be resolved by consensus discussion or a third reviewer will be consulted. Additionally, reviewers should document the rationale for their risk-of-bias assessments, including any limitations or concerns identified during the evaluation process. This transparency helps ensure the reliability and credibility of the scoping review findings.

### Ethical Considerations

Since the scoping review will use published research with deidentified data, no approval from an ethics committee is required. Numerous studies have been conducted on the prevalence of physical capacity in individuals with SCI, but this review is the first to collect and consolidate all these studies. It will also provide policy makers, stakeholders, and health care professionals with epidemiological data to help them in planning health care and policy. The PRISMA-ScR guidelines will be adhered to when publishing the review’s findings in journal articles and academic reports.

## Results

This study was funded in October 2023. Data collection will commence in July 2024, and the results are expected to be published in 2025. A narrative description will be provided for investigations with a low or moderate risk of bias. Clinical heterogeneity will be investigated by examining the characteristics of the study participants, method of diagnosis, and definitions of the cases. If sufficient data are available, subgroup analyses will be conducted for the study population (age, gender, population group, years since injury, level, severity, and type of SCI) and study characteristics (study design, settings, outcome measurement, and the level of physical activity and depression).

## Discussion

### Principal Findings

In this scoping review, we seek to comprehensively analyze the relationship between physical activity and mental health among individuals with SCI. The objective of this study is to comprehensively analyze existing research on the link between physical activity and mental health, identifying the level of physical activity and mental health status, barriers to physical activity, and their impact on psychological well-being in individuals with SCI.

Physical activity plays a crucial role in the mental health of individuals with SCI [29]; however, several barriers to physical activity are associated with mental health. These barriers include the challenges to the management of secondary complications of SCI, which can make recovery complex and difficult [30]. Additionally, individuals with SCI may face mobility limitations and disability, which can hinder their ability to engage in physical activity and negatively impact their mental health [31]. Furthermore, the busy and sedentary lifestyle of modern society can contribute to decreased physical activity levels and an increased risk of depression [31]. It is important to address these barriers and promote physical activity among individuals with SCI to improve their mental health outcomes. It is important to note that further research is needed to fully understand the impact of physical activity on mental health among individuals with SCI.

Building upon our main findings, the scoping review will reveal several noteworthy insights into the relationship between physical activity and mental health among individuals with SCI. First, we expect to observe a positive association between engagement in physical activity and improved mental well-being in this population. Studies would consistently report that regular exercise is correlated with reduced symptoms of depression, anxiety, and stress among individuals with SCI. This finding will align with those of existing literature in the broader context of physical activity and mental health, indicating that similar principles will apply to individuals with SCI.

Furthermore, our review will highlight the multifaceted nature of barriers hindering participation in physical activity among individuals with SCI. These barriers will encompass both internal factors, such as motivation, self-efficacy, and knowledge, as well as external factors, including accessibility, transportation, and financial constraints. Understanding these barriers will be crucial for developing targeted interventions aimed at overcoming these obstacles and promoting physical activity engagement in this population.

Moreover, our findings will underscore the importance of tailored exercise programs that address the unique needs and challenges faced by individuals with SCI. Adaptive exercise equipment, accessible facilities, and personalized exercise regimens will be essential components of effective interventions. Collaborative efforts among health care professionals, rehabilitation specialists, and community organizations will be needed to develop and implement such programs.

Comparing our findings to those in existing literature, our review will corroborate previous studies that have highlighted the beneficial effects of physical activity on mental health outcomes among individuals with SCI. However, it will also extend existing knowledge by identifying specific barriers and challenges that are unique to this population. By elucidating these factors, our review will provide valuable insights for designing targeted interventions and support services tailored to the needs of individuals with SCI.

In summary, our detailed analysis will underscore the importance of promoting physical activity as a means to enhance mental well-being among individuals with SCI. By addressing barriers, developing tailored interventions, and fostering collaborative partnerships, health care professionals and policy makers can work toward improving the overall QOL for this population.

### Limitations

Despite the valuable insights provided by this scoping review, several limitations should be acknowledged. First, the inclusion criteria may lead to the exclusion of relevant studies that do not meet the specified criteria, potentially limiting the comprehensiveness of the review. Additionally, the quality of included studies may vary, leading to potential biases in the synthesis of findings. Furthermore, the reliance on published literature may introduce publication bias, as studies with statistically significant results are more likely to be published. Moreover, the heterogeneity of study designs and methodologies across included studies may pose challenges in synthesizing and interpreting findings. Finally, the scope of the review is limited to studies published in English, which may result in the exclusion of relevant research published in other languages.

### Conclusions

This review of existing studies sheds light on how physical activity relates to mental health in individuals dealing with SCI. Different exercise methods have shown potential in helping individuals who are feeling low, but obstacles such as limited access to facilities and physical challenges still pose significant hurdles. Understanding how exercise affects mood highlights the need for specific plans that address the unique struggles of this group. This emphasizes the importance of personalized approaches to boost mental well-being and enhance the overall quality of life for individuals with SCI. By recognizing the impact of physical activity on mental health outcomes among individuals with SCI, health care providers can prioritize interventions that promote both physical and psychological well-being. Implementing personalized exercise programs tailored to the needs and preferences of individuals with SCI can lead to more effective rehabilitation outcomes and improved QOL. Ultimately, these efforts contribute to a more comprehensive and holistic approach to SCI rehabilitation, fostering greater independence, social participation, and overall well-being for individuals living with SCI.

## Abbreviations

JBI: Joanna Briggs Institute
MeSH: Medical Subject Headings
PRISMA-ScR: Preferred Reporting Items for Systematic Reviews and Meta-Analyses extension for Scoping Reviews
QOL: quality of life
SCI: spinal cord injury

## Data Availability

No data sets were generated or analyzed during this investigation, so data sharing is not applicable. Data sharing will be applicable after the full review study is conducted.
